# High-speed imaging of CNT deagglomeration in aqueous solution with surfactant^☆^

**DOI:** 10.1016/j.ultsonch.2025.107472

**Published:** 2025-07-18

**Authors:** Zhuocheng Xu, Catherine Tonry, Milo S.P. Shaffer, Qianqian Li

**Affiliations:** a Department of Aeronautics, Faculty of Engineering, Imperial College London, London, UK; b School of Computing and Mathematical Sciences, Faculty of Engineering and Science, University of Greenwich, London, UK; c Department of Chemistry, Faculty of Natural Sciences, Imperial College London, London, UK; d Department of Materials Science, Faculty of Engineering, Imperial College London, London, UK; e Department of Mechanical Engineering, Faculty of Engineering, Imperial College London, London, UK

## Abstract

This study investigates the mesoscale deagglomeration mechanisms of multi-walled carbon nanotubes (MWCNTs) in aqueous solutions with and without added surfactant (Triton X-100), using high-speed imaging and numerical simulations. High-speed observations revealed that within the cavitation zone (CZ, defined as the region of high bubble intensity), the addition of surfactant had no obvious effects on deagglomeration behaviour, with most agglomerates remaining intact and only occasional fragmentation events observed. In contrast, in regions outside the CZ, surfactant addition significantly increased the number and stability of microbubble clusters, leading to more frequent interactions with MWCNT agglomerates. Numerical simulations performed under matched experimental conditions confirmed a spatial variation in bubble dynamics, with enhanced microbubble formation and persistence in surfactant-containing solutions, particularly at distances away from the sonotrode. These findings provide direct mechanistic evidence that surfactant not only stabilises dispersed CNTs but also facilitates microbubble-mediated deagglomeration outside the CZ. The results highlight the role of structured bubble activity in extending the effective dispersion region during ultrasonication, offering insight into the optimisation of CNT processing in surfactant-assisted systems.

## Graphical abstract

**Figure:**
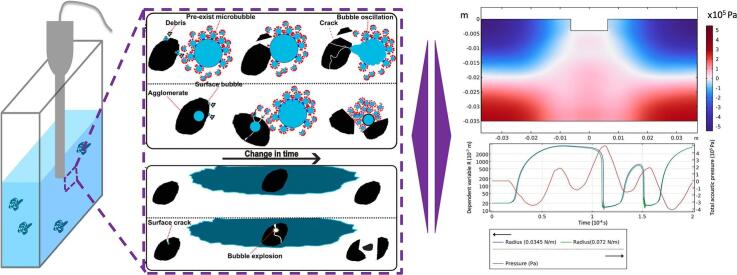

## 1 Introduction

Carbon nanotubes (CNTs) have been extensively studied due to their unique intrinsic properties and broad potential applications, ranging from electronics and sensors to structural and biomedical systems [1]. However, many of these applications require CNTs to be dispersed as individual entities in a liquid medium to fully exploit their properties. Such dispersions are essential as either intermediates for fabricating nanostructured assemblies—such as films, fibres, or networks—or as direct functional forms in applications like biotagging and polymer matrix composites [1,2].

Numerous solvents and shear-based dispersion strategies have been investigated to address CNT agglomeration. Among these alternatives, ultrasonication in aqueous media, in conjunction with amphiphilic agents, has emerged as one of the most widely adopted and effective methods. Amphiphiles used for this purpose include polymers (e.g., polyvinyl alcohol (PVA), poly(m-phenylenevinylene) (PmPV)), biomacromolecules (e.g., DNA, proteins, gum arabic), and specially designed small molecules such as pyrene-functionalised surfactants. Nevertheless, simple and commercially available surfactants, such as sodium dodecyl sulfate (SDS), sodium dodecylbenzene sulfonate (SDBS), Triton X-100, and sodium cholate, remain the most commonly used, due to their consistent dispersibility and low cost [[3], [4], [5]].

The enhancement of CNT dispersion during ultrasonication treatment (UST) through the addition of surfactants is generally attributed to two primary mechanisms: (i) direct adsorption of surfactant molecules onto individual nanotubes [[6], [7], [8], [9], [10], [11], [12]], and (ii) modulation of cavitation bubble dynamics by the presence of surfactants [7,[13], [14], [15], [16], [17], [18]]. In the first mechanism, CNT agglomerates are initially disrupted by the strong shear forces and shockwaves generated during UST. The resulting individualised CNTs are then stabilised against reaggregation by electrostatic or steric repulsion arising from the adsorption of surfactant molecules onto their surfaces [[7], [8], [9], [10], [11], [12]].

Ultrasonication-induced dispersion relies fundamentally on the formation, oscillation, and collapse of cavitation bubbles [14,19], which generate intense local shear fields, microjets, and shockwaves [13,14,[19], [20], [21]]. The dynamics of these cavitation bubbles are primarily governed by the acoustic pressure amplitude and the physicochemical properties of the liquid medium, including surface tension, viscosity, and dissolved gas content [13,15,22]. The addition of surfactants significantly influences cavitation dynamics by modifying several of these parameters. Most notably, surfactants reduce the surface tension of the solution, lowering the energy barrier for bubble nucleation and thereby increasing the number of cavitation events [13,15,22]. In addition, surfactants promote rectified bubble growth [13,16,18,23], enhance bubble nucleation (often associated with surfactant micelles) [14], and retard bubble coalescence [17,18]. These effects result in bubbles exhibiting faster growth rates [13], larger maximum radius [13,15], greater surface morphology instability during oscillation [13,15], and higher micro-jet speeds following secondary collapse [13], compared to surfactant-free solutions. Collectively, these effects result in more frequent and more intense interactions between CNT agglomerates and cavitation bubbles generated by UST.

Previous studies have extensively investigated the improved dispersion of CNTs through the direct adsorption of surfactant molecules, primarily using techniques such as advanced electron microscopy [11]. In contrast, investigations focusing on the dynamic interactions between cavitation bubbles in surfactant-containing solutions and CNT agglomerates, particularly those elucidating bubble-induced deagglomeration mechanisms during UST, remain relatively scarce [20,21,24,25]. In recent years, high-speed imaging has been employed to visualise and analyse the interactions between cavitation bubbles and nanoparticle agglomerates in deionised (DI) water systems [19,20,25]. These studies indicate that deagglomeration mechanisms vary depending on the type and spatial distribution of bubbles, especially in regions proximal and distal to the sonotrode tip. Within the cavitation zone (CZ), near the sonotrode, powerful shockwaves generated by dense clouds of inertial cavitation bubbles dominate the deagglomeration process, leading to the instantaneous rupture or fragmentation of passing agglomerates [20,25]. At greater distances from the CZ, the dominant mechanism shifts to chaotic pulsation of microbubble clusters, which contributes to progressive deagglomeration of CNT assemblies [20,25].

In this study, high-speed imaging was employed to investigate the ultrasonication-induced deagglomeration mechanisms of multi-walled carbon nanotubes (MWCNTs) in aqueous solutions containing Triton X-100 (TX-100), a non-ionic surfactant widely used to enhance CNT dispersion [26,27]. By analysing high-speed image sequences captured at regions proximal and distal to the sonotrode tip, the influence of the surfactant on the dynamic interactions between cavitation bubbles and CNT agglomerates was systematically explored. Additionally, numerical simulations based on the Rayleigh–Plesset equation were conducted to interpret the observed differences in bubble dynamics across various dispersion media [25,28].

## 2 Experimental and methodology

### 2.1 Preparation of dispersion

In this study, 54 mL surfactant-containing solutions were prepared using the non-ionic surfactant Triton X-100 (TX-100, Sigma-Aldrich, UK) dissolved in deionised (DI) water at a concentration three times the critical micelle concentration (CMC) of TX-100 (3 × 0.23 mM) [29]. This concentration was chosen based on our preliminary studies on CNT dispersion within the solutions with different TX-100 concentration (Fig. S1).

### 2.2 Experimental design and data analysis

**Fig. 1:**
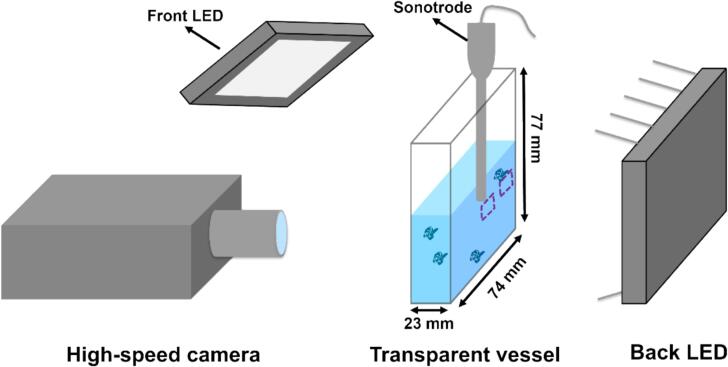
The sketch of the high-speed imaging testing rig used in this study. The regions for high-speed filming were highlight with yellow dotted lines. (For interpretation of the references to colour in this figure legend, the reader is referred to the web version of this article.)

The experimental setup for high-speed imaging was based on a system previously developed in our study of UST-assisted MWCNT deagglomeration in DI water [25]. In the current work, an additional rechargeable LED light source was positioned behind the transparent vessel to reduce shadowing effects from the front illumination (Fig. 1). To investigate deagglomeration mechanisms within and outside the cavitation zone (CZ, defined as the region with high bubble density captured via high-speed imaging) created during UST, two observation regions were selected for high-speed imaging: one directly beneath the sonotrode tip, and another centred at 15 mm laterally from the sonotrode axis, as outlined by the purple dotted line in Fig. 1.

During high-speed imaging, the field of view (FoV), frame rate, and spatial resolution were set to 512 × 384 pixels (approximately 17 mm × 13 mm), 28,000 frames per second (fps), and 34  µm/pixel, respectively. The software packages used for image acquisition and data analysis included AMETEK PCC, ImageJ[30], and MATLAB 2021b. High-speed imaging was repeated twice for each solution condition, and representative frames that most clearly illustrated the underlying deagglomeration mechanisms were selected for presentation.

Ultrasonication was performed using a Sonic VCX 750 ultrasonic processor (Sonic & Materials Inc., USA) equipped with a Ti-6Al-4 V probe (diameter: 12.7 mm; length: 139.0 mm). The system operated at a fixed frequency of 20 kHz. The sonotrode oscillation amplitude and immersion depth were maintained at 48 µm and 10 mm, respectively.

### 2.3 Numerical modelling

The primary difference between the two media, water with and without surfactant, is the surface tension. To compare the effect on the size and number of cavitation bubbles a simple model was developed, solving the Reyleigh-Plesset equation [28], modified to account for the compressibility of water at the pressures used.

(1) $$\rho \left(R\ddot{R}+\frac{3}{2}{\dot{R}}^{2}\right)=p_{\mathrm{gas}}-P_{0}-P\left(t\right)-4\mu \frac{\dot{R}}{R}-2\frac{\sigma }{R}+\frac{R}{c}{\frac{d}{\mathrm{dt}}p}_{\mathrm{gas}}$$

where R(t) is the bubble radius, ρ the density of the water, P_0_ the pressure far from the bubble (assumed to be 1 atm), P(t) the transient liquid pressure, μ the viscosity, σ the surface tension, P(t) the pressure of the liquid surrounding the bubble and p_gas_ the pressure inside the bubble for which we adopt a van de Waals equation of state:

(2) $$p_{\mathrm{gas}}\left(t\right)=\left(P_{0}+\frac{2\sigma }{R_{0}}\right){\left(\frac{R_{0}^{3}-h^{3}}{R^{3}\left(t\right)-h^{3}}\right)}^{\gamma }$$

where R_0_ is the bubble equilibrium radius, h the characteristic hard core Van der Waals radius of the gas, and γ the adiabatic index.

In equation (1), the time derivative of the gas pressure p_gas_ is defined explicitly as:

(3) $$\frac{d}{\mathrm{dt}}p_{\mathrm{gas}}=-3\gamma p_{\mathrm{gas}}\frac{R^{2}}{R^{3}-h^{3}}\frac{\mathrm{dR}}{\mathrm{dt}}$$

These equations were implemented in COMSOL Multiphysics 6.2 using a point ordinary differential equation (ODE) so they could be one way coupled to a transient 2D acoustic solution used to specify P(t). To verify the solver, prior to coupling to the acoustic solver, a sinusoidal pressure was tested against the results in [28] and the same solutions were obtained. The material properties used for this model are shown in Table 1.

**Table 1:** Material properties used for modelling the single bubble behaviours within DI water and solution with added surfactant. The surface tension of these two solutions were determined based on sessile drop test (Fig. S2).

Parameters	Value	Reference
Surface Tension σ (water)	72.1 × 10^−3^ N·m^−1^	Measured in this study
Surface Tension σ (water with surfactant)	34.3 × 10^−3^ N·m^−1^	Measured in this study
Density (water) ρ	1000 kg·m^−3^	User defined
Adiabatic index (water vapour) γ	4/3	[31]
Speed of sound (water) c	1,500 m·s^−1^	[32]
Viscosity (water) μ	1.002 × 10^−3^ Pa·s	[33]
Ambient pressure P_0_	1.00 atm	User defined
Equilibrium radius R_0_	2.0 × 10^−6^*m*	User defined
Hard core radius h	R_0_/8.86	User defined

A two-dimensional geometry representing the experimental vessel (74 mm × 35 mm) was constructed to solve the acoustic field and investigate bubble behaviour under more realistic conditions than those based on a purely sinusoidal wave assumption. Initial acoustic conditions were assumed to be zero pressure relative to ambient (1 atmosphere) in the entire domain. However, acoustic shielding effects due to the presence of bubbles were not included in the model. While such shielding can influence the local acoustic field, our aim was to compare relative bubble behaviour under similar field conditions, rather than to exactly replicate the acoustic environment observed in the experiments.

## 3 Results and discussion

### 3.1 High speed imaging for mesoscale deagglomeration mechanisms

Upon initiation of UST, dispersion of MWCNT powders was observed in both the surfactant-free and surfactant-containing solutions, as indicated by the increasing opacity of the liquid. Comparative analysis of image grayscale levels over the first 30 s of UST (Fig. S1) revealed a more pronounced and rapid decrease in grayscale intensity (less transmitted light) for the surfactant-containing solution, demonstrating enhanced dispersion efficiency in the presence of TX-100.

To explore possible differences in the underlying deagglomeration mechanisms between the two systems, high-speed imaging was used to monitor the dispersion process at two distinct locations: directly within the cavitation zone (CZ) beneath the sonotrode, and in a region further away, referred to indirect interaction with the CZ.

### 3.2 Direct interaction between agglomerates and cavitation zone (CZ)

High-speed imaging conducted directly beneath the sonotrode revealed the majority of MWCNT agglomerates in the TX-100 solution exhibited no noticeable shape changes or fragmentation upon traversing the cavitation zone (CZ). In cases where agglomerate breakage did occur, the deagglomeration mechanisms were completely consistent with our previous observations in DI water [25].

**Fig. 2:**
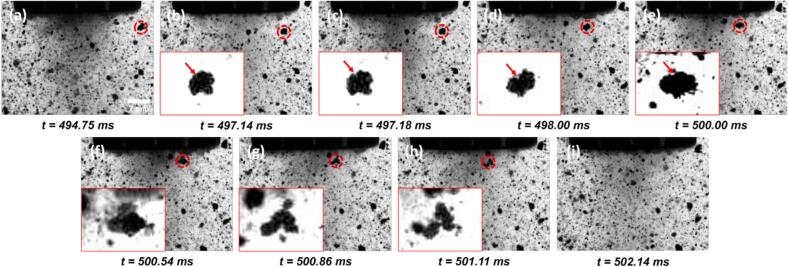
Agglomerate fragmented in the CZ of TX-100 solution during UST. The inset of the figure with the adjusted brightness and contrast shows the zoomed in image of the MWCNT agglomerate labelled by red dotted circle. The red arrows in the figures indicate the positions of defects where the bubble nucleated on the agglomerate. Time values represent the elapsed time after the onset of ultrasonication. (Supplementary Video 1 (SV1)). (For interpretation of the references to colour in this figure legend, the reader is referred to the web version of this article.)

Across all documented deagglomeration events (examples shown in Fig. 2), surface defects, such as cracks, were consistently visible on the agglomerate surfaces prior to their entry into the CZ (indicated by red arrows in Fig. 2). As the agglomerates advanced into the CZ, bubbles were observed to nucleate and oscillate within these pre-existing defects (Fig. 2b–e), further loosening the structure. Eventually, bubble collapse within the CZ, coupled with secondary bubble implosion inside the agglomerates, resulted in their rapid disintegration (Fig. 2f–g), driven by high-speed micro-jets and intense shockwaves [34,35].

We attribute the lack of observable differences in fragmentation frequency and mechanism in surfactant solution primarily to the limited spatial resolution of the high-speed imaging system (34 µm per pixel). At this resolution, variations in bubble population or size within the CZ could not be adequately resolved, thus limiting mechanistic discrimination. In future studies, the use of high-magnification optics is planned to overcome this limitation and enable more detailed analysis of bubble–agglomerate interactions [20].

### 3.3 Indirect interaction with the CZ

#### 3.3.1 Microbubble dynamics in pure water and surfactant solution

**Fig. 3:**
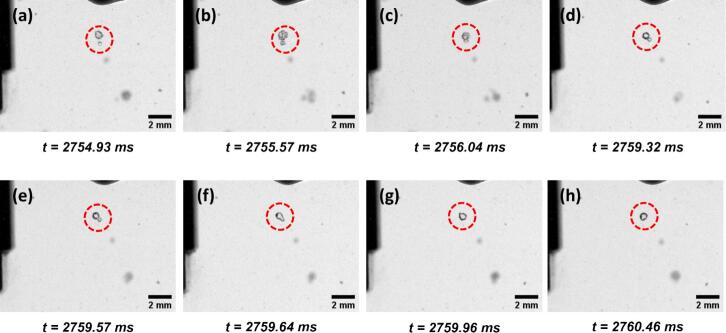
Bubble coalescence in pure DI water during UST. The red circle in each figure indicates the position of an example bubble of interest. Time values represent the elapsed time after the onset of ultrasonication. (For interpretation of the references to colour in this figure legend, the reader is referred to the web version of this article.)

**Fig. 4:**
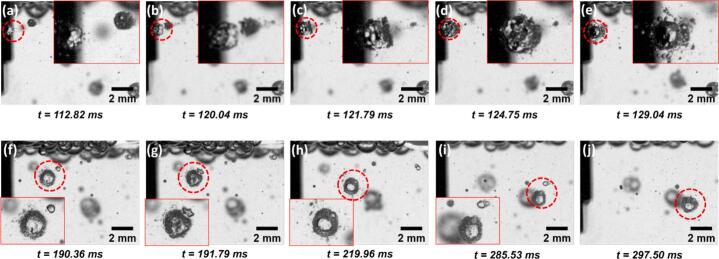
Bubble coalescence in TX-100 solution at 100 % CMC during UST. The inset images in the figures show zoomed-in images of the bubble cluster labelled by red circle with adjusted brightness and contrast. Time values represent the elapsed time after the onset of ultrasonication. (For interpretation of the references to colour in this figure legend, the reader is referred to the web version of this article.)

After examining the direct interactions between the cavitation zone (CZ) and MWCNT agglomerates, our focus shifted to a lateral region adjacent to the sonotrode, where the behaviours of stable bubble clusters were investigated in both DI water and surfactant-containing solutions. In this region, notable differences in bubble dynamics were observed between the two systems, as illustrated by the examples in Fig. 3, Fig. 4, respectively. Specifically, in pure DI water, oscillating bubble clusters were seen to gradually coalesce over time (Fig. 3). In contrast, in the solution containing TX-100 at 300% of its CMC, the attached microbubbles exhibited delayed coalescence, instead forming a larger, more stable bubble cluster (Fig. 4).

**Fig. 5:**
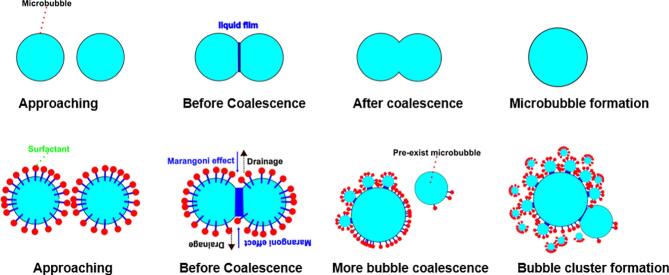
Sketch of bubble coalescence mechanisms in pure water (top) and surfactant solution (bottom).

This observation aligns with previous studies [17,18], which reported that the suppression of bubble coalescence in surfactant-containing solutions is associated with the Marangoni effect, as illustrated schematically in Fig. 5. This effect arises from the non-uniform distribution of surfactant molecules as bubbles approach each other, creating a surface tension gradient between the high-energy inter-bubble interface and the lower-energy bulk solution. The resulting gradient induces a Marangoni flow that opposes film drainage between adjacent bubbles, thereby inhibiting their coalescence [24].

#### 3.3.2 Agglomerate –bubble cluster interaction

**Fig. 6:**
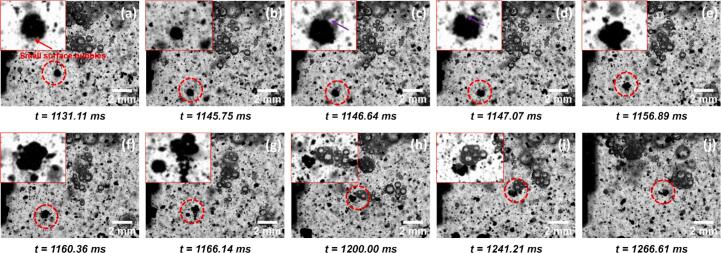
A deagglomeration event captured on the side of sonotrode in TX-100 solution during UST. The inset of each figure with adjusted brightness and contrast shows the zoomed in image of the MWCNT agglomerate labelled by red circle. The red arrow in (a) indicates the position of surface bubbles observed on the surface of MWCNTs. The purple arrows in (c & d) indicate the ‘attacking’ external microbubble. (Supplementary Video 2 (SV2)). Time values represent the elapsed time after the onset of ultrasonication. (For interpretation of the references to colour in this figure legend, the reader is referred to the web version of this article.)

**Fig. 7:**
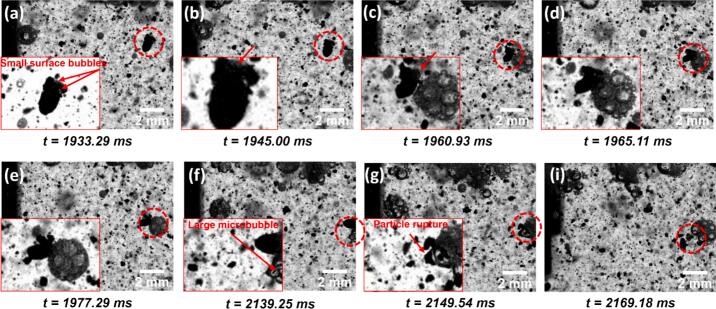
A deagglomeration event captured on the side of sonotrode in TX-100 solution during UST. The inset of each figure with adjusted brightness and contrast shows the zoomed in image of the MWCNT agglomerate labelled by red circle. The red arrow in (a-c) indicates the position of surface bubbles observed on the MWCNT agglomerate. The red arrow in (f) indicate the microbubble that caused final rupture of the agglomerate (g). (Supplementary Video 3 (SV3)). Time values represent the elapsed time after the onset of ultrasonication. (For interpretation of the references to colour in this figure legend, the reader is referred to the web version of this article.)

Following the observation of contrasting bubble behaviours in the presence and absence of surfactant, the influence of TX-100 on MWCNT deagglomeration dynamics was further examined, with representative events summarised in Fig. 6 and Fig. 7. In many cases, deagglomeration was found to be closely associated with the distinct bubble clusters features (Fig. 4, Fig. 5) within the surfactant solution.

In the event shown in Fig. 6, the MWCNT agglomerate exhibited initial surface erosion and loosening, likely caused by small surface-adhered bubbles or trapped air pockets within the agglomerate (Fig. 6a) [20]. Subsequently, the agglomerate was 'attacked' by an oscillating microbubble (Fig. 6c–d), reminiscent of the ‘bubble drone’ behaviour reported by Priyadarshi et al. [20]. This interaction induced intense volumetric oscillation of the agglomerate, leading to internal fragmentation and eventual disintegration (Fig. 6g). The resulting debris formed a trailing “comet-like” structure, similar to features reported in previous studies [20,25]. As the disintegrated agglomerate approached another bubble cluster (Fig. 6h), small bubbles from the cluster migrated into the remaining structure, further widening internal voids and promoting additional fragmentation (Fig. 6i).

In the event illustrated in Fig. 7, multiple small bubbles (indicated by red arrows in Fig. 7a) were observed nucleating within surface defects of an MWCNT agglomerate upon entering the field of view. As UST progressed, these surface-bound bubbles appeared to drive the agglomerate toward a nearby bubble cluster, a motion likely governed by primary and secondary Bjerknes forces [18,36,37] (Fig. 7b–c). During the approach, the surface-adhered bubbles exhibited increasingly vigorous oscillation (Fig. 7b), likely induced by shockwaves generated from the oscillating bubble cluster [20]. Upon attachment, partial rupture of the agglomerate was observed at regions previously weakened by surface bubble activity (Fig. 7c–d). In subsequent UST cycles, the bubble cluster underwent chaotic shape and position oscillations, eventually bringing a large microbubble into proximity with the agglomerate (Fig. 7f). The intense oscillation of this large bubble ultimately triggered complete rupture of the agglomerate (Fig. 7f–g).

### 3.4 Summary of UST induced deagglomeration mechanisms

**Fig. 8:**
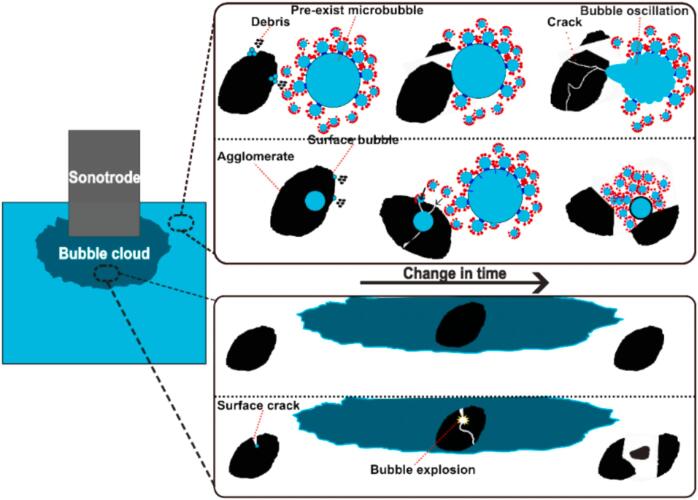
Sketch of the deagglomeration mechanism in TX-100 solution. The position of cine captured was shown on the left-hand side with the region of captured film indicated by the black dotted circle.

The UST-induced deagglomeration mechanisms of MWCNTs in the surfactant-loaded solution, as revealed by high-speed imaging (Fig. 2, Fig. 6, Fig. 7), are summarised schematically in Fig. 8. Within the CZ, due to the limited spatial resolution of the high-speed imaging system, most agglomerates did not exhibit observable fragmentation after passing through the CZ. In cases where fragmentation was detected, the deagglomeration mechanism was consistent with our previous observations in DI water [25], and was primarily attributed to the instantaneous collapse of surface-attached bubbles, triggered by the collapse of surrounding cavitation bubbles within the CZ.

In contrast, compared to DI water, distinct deagglomeration behaviours were observed in regions away from the CZ within the surfactant-containing solution (Fig. 6, Fig. 7), primarily due to the formation of stabilised microbubble clusters. The presence of these clusters increased the probability of agglomerate–bubble interactions and facilitated deagglomeration through several mechanisms: (i) surface erosion and partial rupture of agglomerates during their approach toward bubble clusters (Fig. 6, Fig. 7), (ii) bubble migration into surface defects, which widened internal voids (Fig. 6), and (iii) complete rupture induced by the oscillation and collapse of large bubbles within the cluster (Fig. 7). These observations highlight the enhanced role of structured bubble environments in promoting efficient CNT deagglomeration under surfactant-assisted UST.

### 3.5 Results from numerical analysis

To further examine the enhanced bubble dynamics and increased bubble number density, particularly in regions indirectly influenced by the cavitation zone (CZ), numerical simulations were conducted using parameters aligned with the high-speed imaging conditions (summarised in Table 1).

**Fig. 9:**
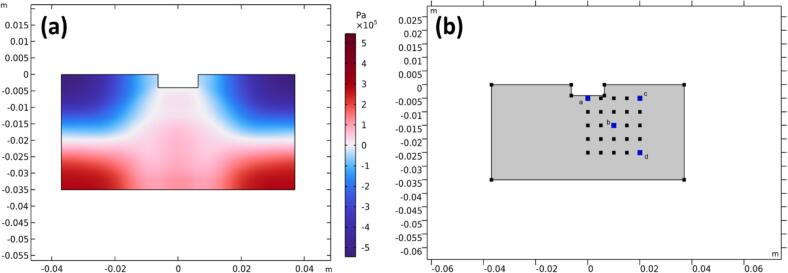
(a) Computed acoustic pressure distribution at 1.5 × 10^−4^ s after the initiation of ultrasonication. (b) Selected positions (a–d) used for bubble radius analysis shown in Fig. 10.

**Fig. 10:**
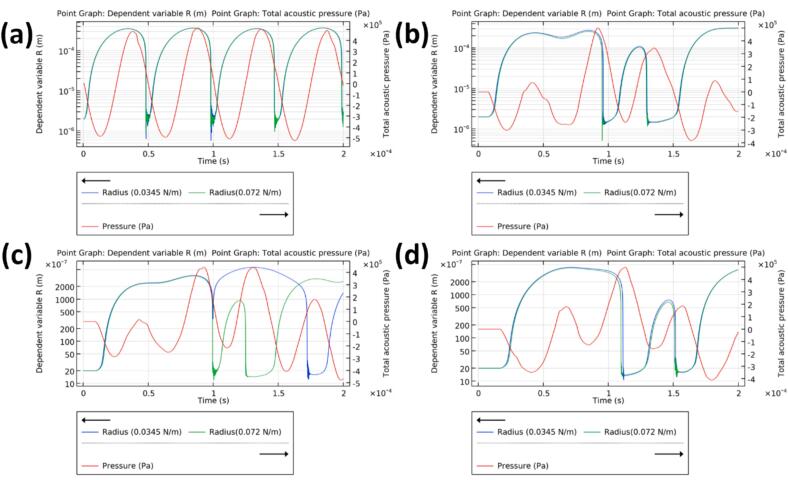
Variation in computed acoustic pressure and bubble radius in solutions with (green) and without (blue) surfactant at locations (a)–(d) as indicated in Fig. 9b. (For interpretation of the references to colour in this figure legend, the reader is referred to the web version of this article.)

Fig. 9a shows the computed acoustic pressure field at 1.5 × 10^−4^ s after the initiation of ultrasonication. At this time, the acoustic waves have propagated throughout the simulation domain. Beyond this point, the maximum pressure in the domain stabilises, corresponding to approximately four complete sonotrode oscillation cycles. Fig. 10 presents the spatial distribution of calculated bubble radii at four representative locations: 0.00 mm (a), 14.14 mm (b), 20.00 mm (c), and 28.28 mm (d) from the sonotrode tip, as indicated in Fig. 9b. These positions were selected to assess bubble dynamics both near and further from the sonotrode, enabling spatial analysis and direct comparison with high-speed imaging observations.

At positions (a) and (b), the maximum bubble radii in both DI water and the surfactant-containing solution were comparable, reaching approximately 3.5 × 10^−4^ mm and 3.0 × 10^−4^ mm, respectively. This observation aligns with experimental findings that showed no significant difference in deagglomeration mechanisms near the cavitation zone (CZ). However, notable differences in bubble behaviour emerged at locations farther from the sonotrode, particularly at 20.00  mm (Fig. 10c). While initial bubble growth was similar in both solutions, the second bubble in the surfactant-containing medium reached a smaller maximum radius and collapsed more rapidly than its counterpart in DI water.

These findings suggest that bubbles form more readily and collapse more rapidly with slightly smaller radius variation in the surfactant-containing solution, consistent with its lower surface tension and in agreement with previous simulation studies [13,15]. As the bubble-induced shear forces correlate with the amplitude of bubble radius oscillation [38,39], we infer that the improved deagglomeration efficiency outside the CZ, observed via high-speed imaging, is primarily driven by more frequent but slightly less intense bubble–agglomerate interactions during UST. It is worth noting that some previous studies [13,15] have reported that, under prolonged ultrasonication, the difference in bubble radius in fluids with and without surfactant may be more significant due to rectified diffusion [23]. As the simulation in our current study is focused on early-time dynamics, further experimental and computational investigations are needed to clarify the long-term evolution of bubble clusters and their cumulative effects on deagglomeration in these regions.

These simulation results show how surfactant addition influences bubble dynamics across spatial regions. In particular, the enhanced bubble generation observed at locations distal to the sonotrode suggests that surfactants can effectively extend the operational range of deagglomeration beyond the primary cavitation zone. Moreover, the simulation also offers perspective on how surfactant concentration affects nanoparticle deagglomeration efficiency during UST. Specifically, at concentrations below the CMC, increasing surfactant levels progressively reduce surface tension [40,41]; the associated enhanced acoustic activity in regions distal to the CZ would be expected to increase deagglomeration efficiency. Above the CMC, further increases in concentration have negligible effect on surface tension, and thus little additional influence on cavitation dynamics or deagglomeration efficiency. This interpretation is supported by our grayscale analysis in Fig. S1, which shows that increased surfactant concentration below the CMC correlates with a more rapid drop in grayscale intensity during UST.

In future work, the accuracy of the numerical model will be further validated through direct comparison with experimental results under varying UST parameters, such as container volume, sonotrode positioning, oscillation amplitude, and pulse modulation. Further validation of the parameter space will allow the model to be extended and refined to account for scale-dependent effects, enabling improved prediction and optimisation of cavitation activity in industrial-scale processing environments.

## 4 Conclusion

Ultrasound treatment (UST) is widely used to disperse multi-walled carbon nanotubes (MWCNTs), with surfactants commonly added to stabilise the resulting suspensions. In this study, high-speed imaging and numerical simulations were employed to investigate how surfactant addition not only enhances dispersion stability but also modifies the underlying deagglomeration mechanisms.

Within the dense bubble cloud directly beneath the sonotrode, i.e. the cavitation zone (CZ), the presence of surfactant had minimal effect on dispersion behaviour; most agglomerates passed through unaltered, with only occasional fragmentation events linked to the collapse of inertial cavitation bubbles. In contrast, outside the CZ, the addition of surfactant significantly increased the formation and stabilisation of microbubbles, which accumulated into clusters. These clusters played a central role in promoting MWCNT deagglomeration through multiple pathways, including enhanced oscillation of surface-bound bubbles, widening of internal gaps via bubble migration into surface defects, and complete rupture induced by the collapse of larger bubbles within the clusters.

Numerical simulations supported these experimental observations by showing that bubbles form more readily and collapse more rapidly in the surfactant-containing solution, particularly at locations distant from the sonotrode (e.g., 20  mm). This enhancement in cavitation activity results in a greater number of microbubbles and clusters, thereby increasing the likelihood of bubble–agglomerate interactions—consistent with the enhanced deagglomeration efficiency observed in high-speed imaging experiments.

Together, these findings demonstrate that surfactants not only stabilise dispersed MWCNTs but also actively facilitate deagglomeration by enabling spatially extended, microbubble-mediated mechanisms beyond the primary cavitation zone.

## CRediT authorship contribution statement

**Zhuocheng Xu:** Writing – original draft, Methodology, Investigation, Formal analysis, Data curation. **Catherine Tonry:** Writing – review & editing, Software, Investigation, Formal analysis, Data curation. **Milo S.P. Shaffer:** Writing – review & editing, Supervision, Resources, Conceptualization. **Qianqian Li:** Writing – review & editing, Validation, Supervision, Resources, Investigation, Conceptualization.

## Declaration of competing interest

The authors declare that they have no known competing financial interests or personal relationships that could have appeared to influence the work reported in this paper.
