# Supplementary material for: High-speed imaging of CNT deagglomeration in aqueous solution with surfactant
Source: Ultrason Sonochem. 2025 Jul 18;120:107472. doi: 10.1016/j.ultsonch.2025.107472 (PMC12312057; doi:10.1016/j.ultsonch.2025.107472)
Supplement: Supplementary Data 1 [file mmc1.docx]

**High-speed imaging of CNT deagglomeration within aqueous solution**

*Zhuocheng Xu^1^, Catherine Tonry^2^, Milo S. P. Shaffer^3,4^, Qianqian Li^1*^*

1. *Department of Aeronautics, Faculty of Engineering, Imperial College London, London, UK*
2. *School of Computing and Mathematical Sciences, Faculty of Engineering and Science, University of Greenwich, London, UK*
3. *Department of Chemistry, Faculty of Natural Sciences, Imperial College London, London, UK*
4. *Department of Materials Science, Faculty of Engineering, Imperial College London, London, UK*

**Corresponding author. Email address:* [*qianqian.li@imperial.ac.uk*](mailto:qianqian.li@imperial.ac.uk)

Greyscale analysis for optimum surfactant concentration determination:


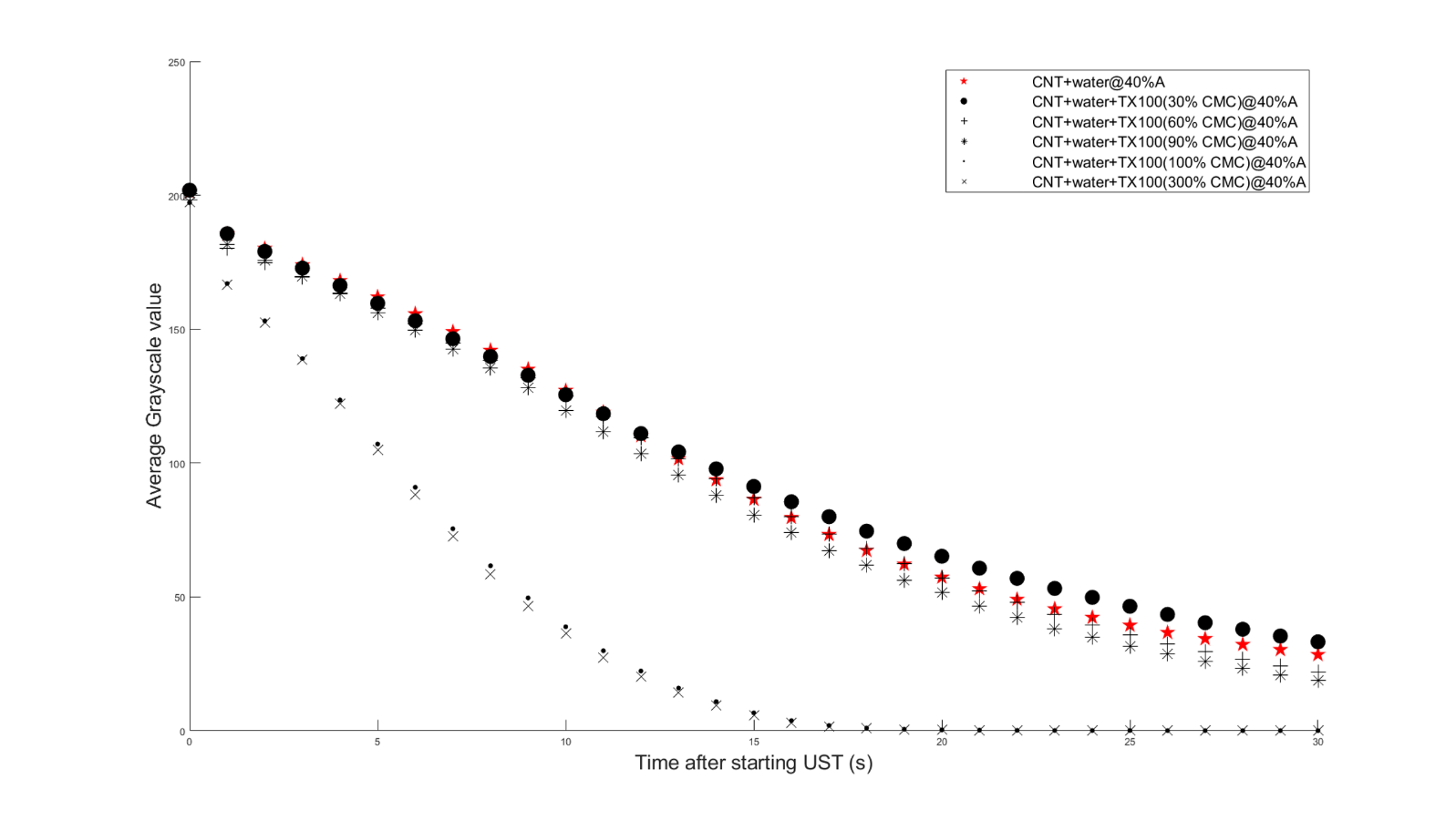


*Figure S1 Change in average Grayscale value during the UST of different solutions.*

To study the effect of surfactant concentration on dispersion efficiency, the changes in average Grayscale value during UST with different surfactant concentration were calculated and presented shown in *Figure S1* above. To minimize any effects caused by reflection, the LED bulb in the front of the vessel was switched off when capturing these images. From the plot, an abrupt change in average Grayscale value (hence the dispersion effectiveness) was observed when increasing the surfactant concentration above 1 CMC.

Sessile drop test for surface tension measurements:

The values of surface energy in DI water and solution with 300% CMC of TX-100 was determined by using sessile drop methods shown in Figure S2. Based on the measurement, the surface energy for DI water and the surfactant solution were determined as 72.14 mN/m and 34.29 mN/m respectively.


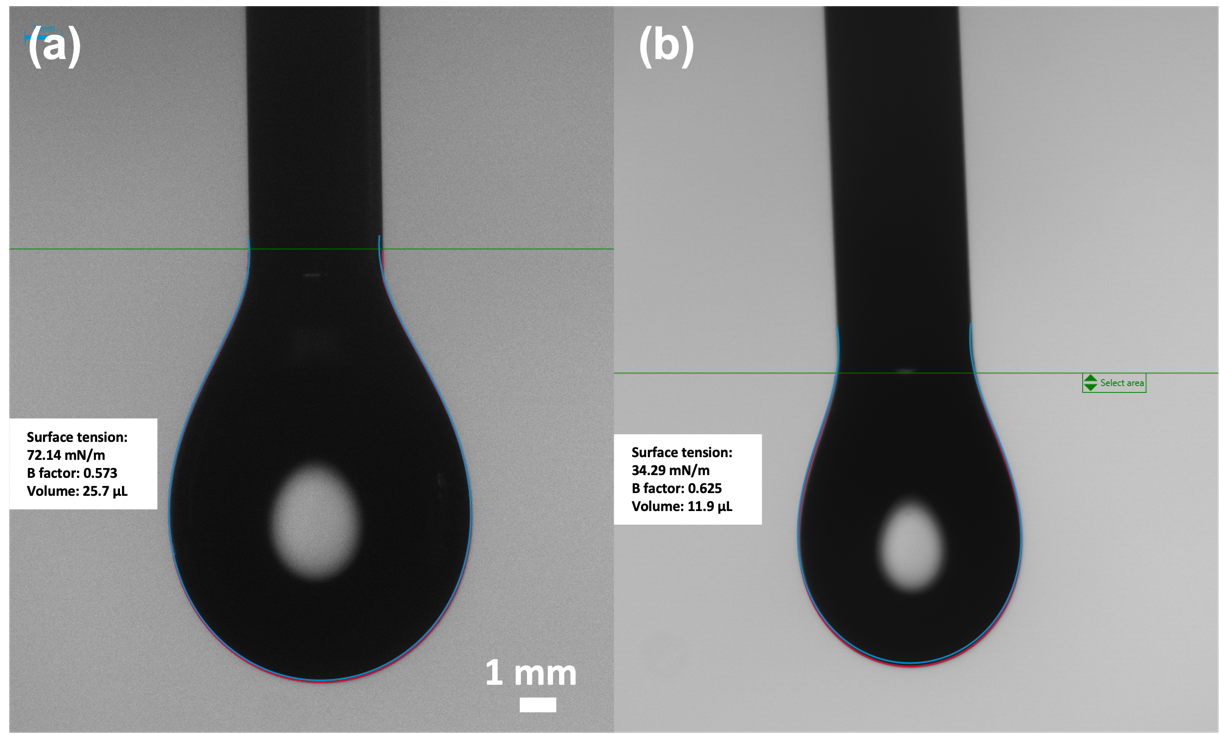


Figure S2 The sessile drop test for pure DI water (a) and solution with 300% CMC of TX-100 (b).
